# Practical Notes and Formulæ

**Published:** 1884-04-20

**Authors:** 


### PRACTICAL NOTES AND FORMULÆ.

**To Produce Hilarity.**—The following formula is claimed, by a writer in a French journal, to produce a marked exhilarating effect upon females, causing, in some instances, excessive laughter:

℞ Tinc. Ergot.................................*m* 75.
Sol. phos. soda (10 per ct.).......................*m* 225.

Mix in about two ounces of sweetened water and give at a single dose on an empty stomach.

The effect of this dose upon a male is said to cause a severe headache.

**Treatment of Typhoid Fever.**—Dr. Q. C. Smith, in Journal American Medical Association, says: For several years I have used a prescription for typhoid fever somewhat as follows, and have been much pleased with it. Almost all of my typhoid fever patients recover, though I have no hospital practice.

℞ Sugar,

Gum arabic, } aa.............................ʒ j,

Spts. turpentine,

Syr. ipecac.................................ʒ ij,

Iodide sodium .................................gr. x,

Cinnamon water, q. s. ft........ .... ............℥ ij.

ft. sol.........................................

S. Teaspoonful every two to four hours.

Sponging with warm or cold water is grateful. Small doses t d. of quinine in some cases, sub. nit. bismuth and salicine to check diarrhœa, about cover the drugs used.

Good soups, fresh fruit juices, as from baked apples and their well-cooked pulp, and orange and lemon juices, and sometimes liquid lactopeptine t. d. after nourishment, a teaspoonful. Fruit juices are too much neglected in fevers.

Change body linen every twenty-four or forty-eight hours; bed linen every forty-two to seventy-two hours.

**German Anti-Spasmodic Mixture.**—The following prescription is taken from the German pharmacopœia, and is much used as an anti-spasmodic in hysteria.

From fifteen minims to a half-drachm or more may be administered in a potion, or by enema two or three times daily.

The dose may be increased when the convulsive accidents are of great intensity:

℞ Tinct. asafœtidæ.............................℥ ss,

Tinct. castorei.................................℥ ss,

Tr. opii.......................................ʒ j.

M.

—*Med. and Surg. Rep.*

**A New Injection for Gonorrhœa.**—This sedative and antiseptic injection may be used even in the acute stage with good results. It is claimed to be superior to any other single injection:

℞ Pulv. iodoformi .................................20,
Acidi carbolici.....................................10,
Glycerini.............................................80,
Aquæ destillatæ...................................200.
M. —*Campana.*

**Formulas.**—Dr. Q. C. Smith sends the following formulas to Texas Courier of Medicine:

Gonorrhœa Injection—

℞ Bichloride hydrag...........................gr. ¼,

Mucilage acacia..................................℥ j,

Aqua dist. qs. ft...................................℥ iv.

M. Ft. sol.

S. Two syringefuls just after each urination.

Deodorized Iodoform—

℞ Iodoform, in fine pow.der.....................dr. ij,

Tanic acid...........................................dr. j,

Balsam Peru,

Oil sassafras,

Oil roses,

Oil camphor,

Oil eucalyptus, } ..........................aa gtt. drops iv,

M. Ft. powder.

S. Put in vial.

Gonorrhœa injection—

℞ Mur. hydrastina,

Chloral hydrate,

Sulph. quinine,

Sulph. morphia, } ..........................aa gr. j,

Aqua dist. qs. ft...........................qs. ft. ℥ viii,

M. Ft. sol., add:

Sulphurous acid...............................dr. j,

M. Ft. sol.

S. Two syringefuls just after each urination.

**Chloride of Zinc in Gonorrhœa.**—Lloyd's choice of chloride of zinc in gonorrhœa was based on his conception of the affinity it has for albumen. His formula:

℞ Zinci chloridi.....................................gr. j,
Aquæ puræ..........................................℥ j.
M. Sig. To be used as an injection every eight hours. In addition the perineum and penis are to be bathed frequently with tepid water.

We use the injection as often as six times a day, directing the patient to micturate before each injection.

